# Differential pathogenesis of intracerebral and intramuscular inoculation of street rabies virus and CVS-11 strains in a mouse model

**DOI:** 10.22038/ijbms.2021.54264.12188

**Published:** 2021-07

**Authors:** Firozeh Farahtaj, Leila Alizadeh, Alireza Gholami, Mohammad Sadeq Khosravy, Rouzbeh Bashar, Safoora Gharibzadeh, Hamid Mahmoodzadeh Niknam, Amir Ghaemi

**Affiliations:** 1 National Center for Reference & Research on Rabies, Institut Pasteur of Iran, Tehran, Iran; 2 Department of Virology, Pasteur Institute of Iran, Tehran, Iran; 3 Viral vaccine Production, Pasteur Institute of Iran, Karaj, Iran; 4 Department of Epidemiology and Biostatistics, Research Center for Emerging and Reemerging of Infectious Diseases, Institut Pasteur of Iran, Tehran, Iran; 5 Immunology Department, Pasteur Institute of Iran, Tehran, Iran

**Keywords:** Astrocyte, Intracerebral, Intramuscular, Microglia cells, Neurons, Street rabies virus

## Abstract

**Objective(s):**

The mechanisms of rabies evasion and immunological interactions with the host defense have not been completely elucidated. Here, we evaluated the dynamic changes in the number of astrocytes, microglial and neuronal cells in the brain following intramuscular (IM) and intracerebral (IC) inoculations of street rabies virus (SRV).

**Materials and Methods:**

The SRV isolated from a jackal and CVS-11 were used to establish infection in NMRI-female mice. The number of astrocytes (by expression of GFAP), microglial (by Iba1), and neuronal cells (by MAP-2) in the brain following IM and IC inoculations of SRV were evaluated by immunohistochemistry and H & E staining 7 to 30 days post-infection.

**Results:**

Increased numbers of astrocytes and microglial cells in dead mice infected by SRV via both IC and IM routes were recorded. The number of neuronal cells in surviving mice was decreased only in IC-infected mice, while in the dead group, this number was decreased by both routes.

The risk of death in SRV-infected mice was approximately 3 times higher than in the CVS-11 group. In IC-inoculated mice, viral dilution was the only influential factor in mortality, while the type of strain demonstrated a significant impact on the mortality rate in IM inoculations.

**Conclusion:**

Our results suggested that microglial cells and their inflammatory cytokines may not contribute to the neuroprotection and recovery in surviving mice following intracerebral inoculation of SRV. An unexpected decrease in MAP2 expression via intramuscular inoculation indicates the imbalance in the integrity and stability of neuronal cytoskeleton which aggravates rabies infection.

## Introduction

Rabies is acute viral encephalitis, responsible for approximately 59000 deaths and more than 3.7 million disability-adjusted life years lost annually (1). During the incubation period (2-4), the virus replicates at the site of inoculation (5), unless inactivated by natural or induced host immune responses. The time required for the virus to replicate in the striated muscle can contribute to a longer incubation period (2, 3). 

Without any viremia, the virus can enter the peripheral nerves, travel to the spinal ganglia through retrograde axonal transport (6), and finally reach the brain (7) at a rate of about 50–100 mm per day (8, 9), leading to neuronal dysfunction and death (10). Neurotracing studies showed an exclusive motor pathway in the spread of the rabies virus to the spinal cord following intramuscular (IM) inoculation in rats and primates (11, 12). The highest viral concentration can be found in the brainstem, basal ganglia, hippocampus, and cerebellum (7, 13). The symptoms of rabies are associated with minimal neuropathological findings, which produce low inflammatory and microglial reactions in the central nervous system (CNS) (10). The role of astrocytes, which constitute the most abundant cells in the brain, has been reported in the induction of innate immunity by producing interferon (IFN) (14) and increasing the blood-brain barrier permeability (15) as well as inflammation (16). Moreover, reactive astrocytosis has been associated with immunohistochemical detection of glial fibrillary acidic proteins (GFAPs) in different conditions (17).

The role of viral replication in microglia and astrocytes in the dissemination and persistence of infection in the host brain has been also suggested (18, 19). The importance of astrocyte infection with both wild-type and attenuated rabies viruses has been also discussed during rabies virus infection (20). A specific protein, known as microtubule-associated protein 2 (MAP2), which is highly expressed in mature neurons, shows exclusive increase, following inoculation with fixed and street rabies virus (SRV) strains (21).

The rabies virus cannot be cleared by innate or adaptive immune responses following the emergence of clinical symptoms (22); although the mechanisms of resistance or survival remain unclear (23). Factors involved in different pathologies may include different rabies strains, routes of infection, immune status of the host, and susceptibility to the rabies virus (24). Regardless of age and type of viral strain, mice are more susceptible to the IM route of infection, compared with subcutaneous or intraperitoneal routes (25).

Inflammatory and microglial reactions in the nervous system (26) do not seem to induce major neuronal loss (27). Therefore, the aim of this study was the use of other alternative markers of morphological changes to study the dynamics of cellular infection in the brain. We investigated the changes that occurred in the cellular number of immune responses in the CA1 layer of the hippocampus of the brains of mice following intramuscular and intracerebral injection of SRV isolated from a jackal in Iran. The other objective was to determine the impact of different routes of inoculations on the expression of particular resident cell types in the brain stem in order to obtain more information about immune responses in this privileged area. 

## Materials and Methods

**Figure 1:**
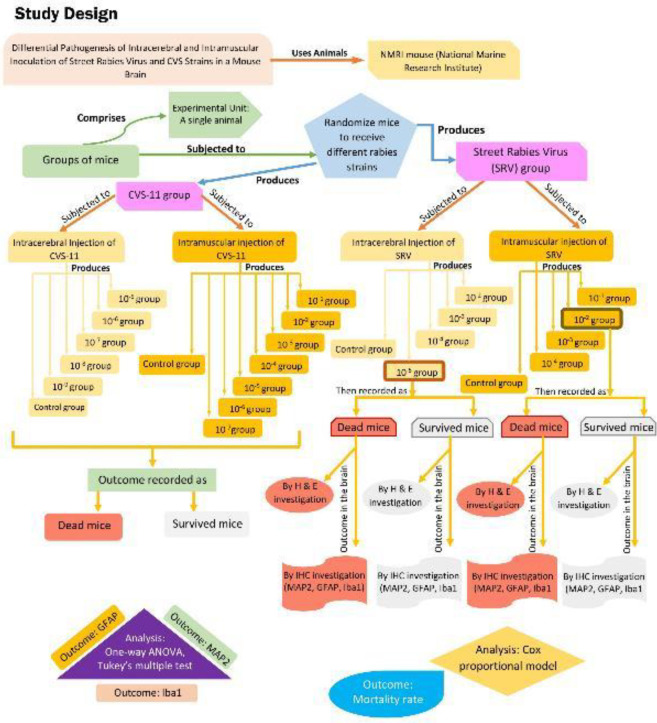
A study plan demonstrates the impact of the type of strain (SRV and CVS-11) on the survival of the mice and also illustrates the effects of IM and IC inoculations of SRV on immune cellular (astrocyte, microglia, and neurons) responses in the brain tissue. For the measurement outcome of IHC and histopathological results, a quantitative analysis was performed on detailed panoramic images of samples in each test group using the Image J software package. One-way analysis of variance (non-parametric) and Tukey's multiple comparison tests were performed for data analysis. Cox proportional analysis was used for survival analysis of the effect of type strain on the survival of the mice

Two data sources were used in this study: One illustrates the SRV-histopathological changes in cellular immune responses in the CA1 layer of the hippocampus of the brain. The other represents the outcomes resulting from CVS-11 infection compared with SRV via IM and IC injection in mice (Figure 1).

***Rabies viruses, dilutions, and laboratory animals***

***A w***ild-type (SRV) (AC:KX148186) isolated from a jackal and CVS-11 strains were obtained from the viral collection of the Centre for Reference and Research on Rabies of Pasteur Institute of Iran. 

As shown in Table 1, Serial 10-fold dilutions of CVS-11 strain (with the intracerebral LD_50_/dose of 6.518, and intramuscular LD_50_/dose of 1.714) and SRV strain (with the intracerebral LD_50_/dose of 5.274, and intramuscular LD_50_/dose of 2.400) were prepared.

One hundred and ninety 4-6-week old female NMRI (National Marine Research Institute) mice with a weight range of 18–24 g were purchased from Karaj Laboratory Animal Centre of Pasteur Institute of Iran. 

One hundred and eighty mice were randomly divided into eighteen groups (10 mice/group) and the remaining ten mice were randomly assigned to two groups (5 mice/group). 

Then, five groups of 10-mice receiving 10-fold dilutions of CVS-11 (starting with 10^-5^ to 10^-9^) and four groups of mice receiving the SRV stock (starting with 10^-3^ to 10^-5^) were challenged by the IC route (0.03 ml of each dilution). Since the cut-off death for the two strains was different, we used different dilutions for each route of inoculation (28). For intracerebral inoculation, mice were anesthetized with diethyl ether (100921 Merck) before injection of 0.03 ml/mouse. For intramuscular inoculation 0.1 mL per dose of SRV viral dilutions (10^-1 ^to10^-4^) and CVS-11 (10^-1 ^to10^-6^) were injected into the hind limb muscle (Table 1).

For each group, inoculation of sterilized PBS under similar conditions was used as a sham group.

All experiments were performed in BSL-2, according to the guidelines of the Ethical Committee of Pasteur Institute of Iran (ethics number: et- 91/0201/4549).

The mice were monitored daily for 30 days, beginning with the first post-inoculation day. 

Death from rabies was confirmed by direct fluorescent antibody testing (FAT). At the end of the study, the surviving mice were euthanized by CO_2_. The brains of mice were removed from the skull and kept in a fresh solution of 10% neutral buffered formalin at 4 °C until processing for immunohistochemistry and H & E.

***Immunohistochemistry***

Immunohistochemistry was performed on the brain stem of two IC groups inoculated with SRV (dilution of 10^-5^) and two IM groups (10^-2^ dilution) according to the manufacturer’s protocol (DAB KL5007, Biopharmadex) and using antibodies against a series of markers including rabbit polyclonal antibody to ionized calcium-binding adaptor molecule 1 (Iba1 for microglial cells) (Abcam 153696, 1:100); rabbit polyclonal antibody to GFAP (GFAP for astrocytes) (Abcam 7260, 1:5000); and mouse monoclonal antibody to MAP-2 (MAP-2 for neuronal cells) (Abcam 11268, Clone AP-20, 1:200). 

***Histological and digital image analysis***

Quantitative analysis was performed on detailed panoramic images of samples in each test group using the Image J software package. A Zeiss-Ntzmiiier microscope with a 1 mm^2 ^mesh in a 10X light microscope eyepiece and a 40X objective lens was used to locate the area. One-way analysis of variance (non-parametric) and Tukey’s multiple comparison tests were performed for data analysis.

## Results

***Effects of different types of virus and routes of inoculation on rabies mortality rate***

Out of 180 samples, 110 mice were infected by CVS-11, and the remaining 70 received SRV inoculation. A total of 69 mice had died at the end of the study. The mortality and survival of the infected mice were analyzed by Cox proportional hazard model. The mortality rate corresponding to SRV (62.34%) was higher than that of CVS-11 (19.09%, *P*<0.001). Regardless of routes of inoculation and viral dilutions, the mortality risk in the SRV group was 3.8 times higher than CVS-11 (*P*<0.0001, CI: 2.28-6.38).

In mice with the IC route of inoculation, the type of strain had no significant effects on mortality caused by SRV and CVS-11, and the viral dilution was the only influential factor in the mortality of mice (*P*<0.05). In contrast, in the IM group, the type of strain showed a significant effect on the mortality rate (Table 1). 

As shown in Table 2, the mean incubation period following intramuscular inoculation was 11.7 days for SRV (CI: 10.48-12.94) versus 6.2 days for CVS-11 (CI: 5.82-6.49). Intracerebral inoculation of SRV and CVS-11 indicated 8.6 (CI: 7.97-9.14) and 5 (CI: 5-5) days of incubation, respectively (*P*<0.05). 

***Effects of SRV infection on expression of GFAP, IbaI, and MAP2 in brainstem via different routes of inoculation***

***Histopathological findings***

**Figure 2:**
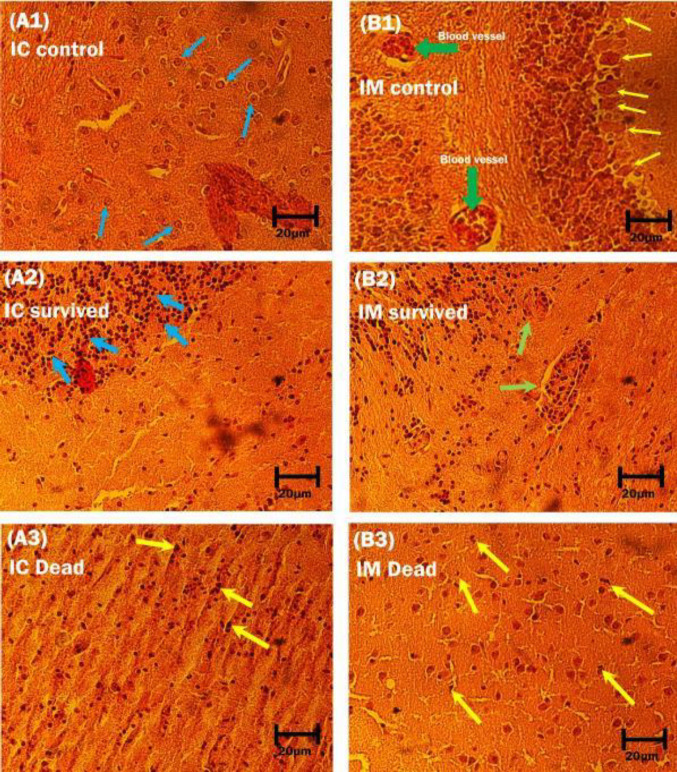
Histopathological findings in brain stem slices prepared from experimentally SRV-infected mice. (A1): heterogeneous neuronal populations within the brain in the intracerebral control group (blue arrows). (B1): a single layer of large-sized projection neurons (Purkinje neurons for the cerebellum (yellow arrows) and also cross-sections of blood vessels containing red blood cells without inflammatory cells (green arrows). (A2): a view of cellular infiltration which is (predominantly) composed of lymphocytes; were seen throughout the brain in IC surviving mice (thick blue arrows). (B2): in IM survived, mixed mononuclear inflammation and also babes-like nodules (green arrows), which is an indicator of pathologic lesions in rabies consisting of glial cells. (A3) and (B3): a pattern of dark neurons within the layer of the hippocampus (thick yellow arrows) in the IC and IM dead groups. H&E, 40X

The panoramic microscopic view of histological preparations via H & E staining indicated a view of inflammation mainly composed of lymphocytes in neuropils of IC-surviving mice (Figure 2: A2). Moreover, infiltration of lymphocytes, a pattern of perivascular cuffing of mononuclear cells, and Babes-like nodules, consisting of glial cells, were observed in surviving mice from the IM group (Figure 2: B2). Apoptotic-like features (nuclear pyknosis and shrinkage of cytoplasm) in the neuronal cells and lymphocytes, resembling dark cells were also observed in dead mice of both IC and IM groups (Figure 2: A3 and B3).

***Immunohistochemical findings***

*Astrocytes*

**Figure 3:**
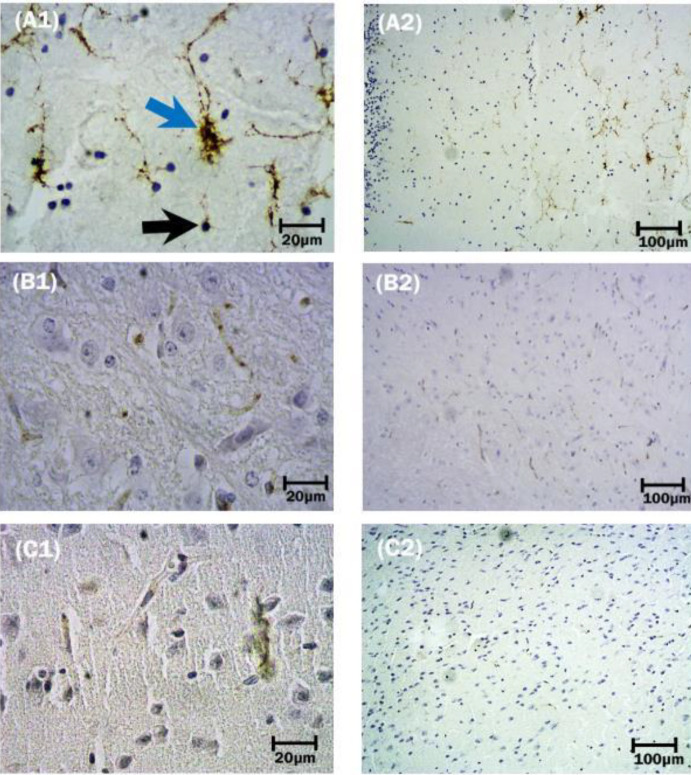
Immunoreactivity of astroglial cells by anti-GFAP antibody following intracerebral and intramuscular inoculation of street rabies virus (SRV) in the surviving group. A1: Cellular processes of reactive astrocyte (blue arrow) show signs of hypertrophy, become thicker, and therefore visible over a longer distance when visualized with an antibody against GFAP. Black nuclei with irregular and pale cytoplasm show a necrotic cell (black arrow). A2: Intracerebral inoculation of the virus shows astroglial cells of protoplasmic shape. B1and B2 show astrocytes and intact neurons in the control group of mice. In C1 and C2, GFAP expression of astrocytes following intramuscular inoculation of SRV is shown. Immunohistochemistry, 10X and 40X

**Figure 4:**
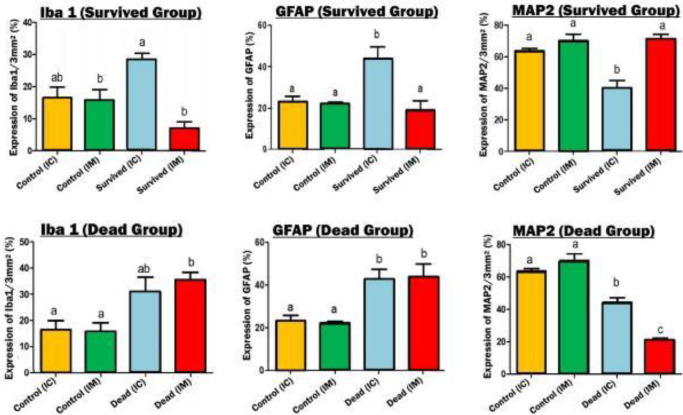
Statistical analysis of MAP2 (neuronal marker), GFAP (astrocyte), and Iba1 (microglial cell) expression in dead and surviving mice inoculated by intracerebral (IC) and intramuscular (IM) inoculations of street rabies virus. Data were analyzed (a: *P*<0.05, b: *P*<0.001, c: *P*<0.0001) and compared with each control group

Hypertrophy and proliferation of astrocytes were observed in the IC-inoculated mice (Figure 3: A2). Astrocytes showed cytoplasmic enlargement associated with the generation of new and thicker processes (Figure 3: A1), in addition to the further accumulation of GFAP content, resembling reactive astrocytosis. As shown in Figure 3: (A1), astrocytes were found near neurons, although they could be found anywhere within the neuropil. There was also a significant increase in the GFAP expression of astrocytes in the surviving IC group versus IM-inoculated mice (*P*<0.05). Among the dead group, no significant difference was found in the number of astrocytes between IC and IM groups (*P*<0.0052), nevertheless they showed a marked increase compared with their corresponding control groups (*P*<0.05) (Figure 4).

*Microglial cells*

**Figure 5:**
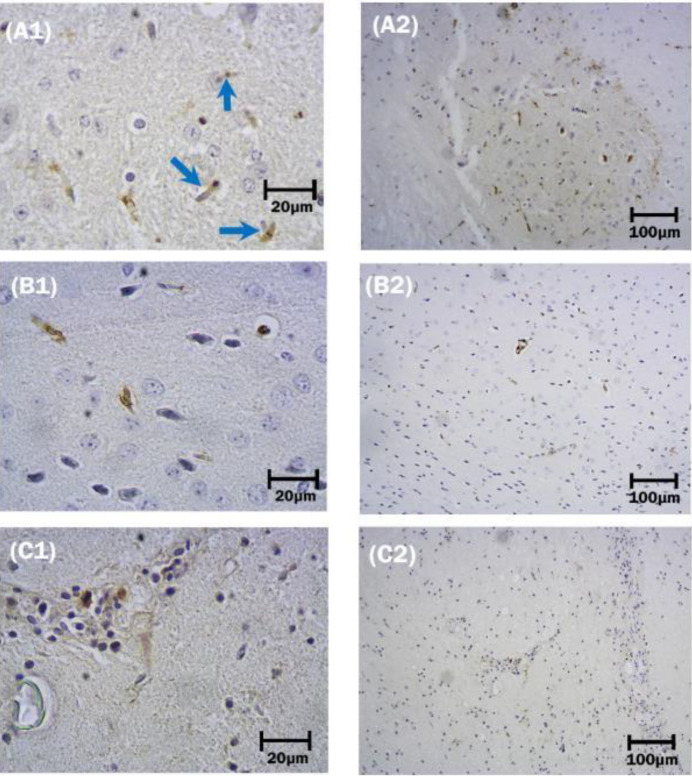
Immunoreactivity of microglial cells by anti-Iba1 antibody following inoculation of street rabies virus. (A1 and A2): small rod-like cells (blue arrows) observed in the slices prepared from the brain stem of surviving mice injected by intracerebral route. (B1 and B2): control group of mice inoculated by intracerebral PBS-inoculation. (C1 and C2): microglial cells in surviving mice injected by intramuscular route. Immunohistochemistry; 10X and 40X

Transformation of small rod-like cells into reactive amoeboid microglia rarely found in Iba1-immunolabeled sections (Figure 5: A1 and A2). In surviving mice, a higher number of microglial cells in the brain inoculated by the IC route was demonstrated than in IM-infected mice; although they did not show any significant differences with their corresponding controls by statistical analysis(*P*<0.005) (Figure 4). In contrast, in the dead group, a significantly increased number of microglial cells in IM and IC inoculated mice was demonstrated and statistical analysis revealed no significant difference with the two routes of inoculation (*P*<0.0166) (Figures 4 and 5). 

*Neuronal cells*

**Figure 6:**
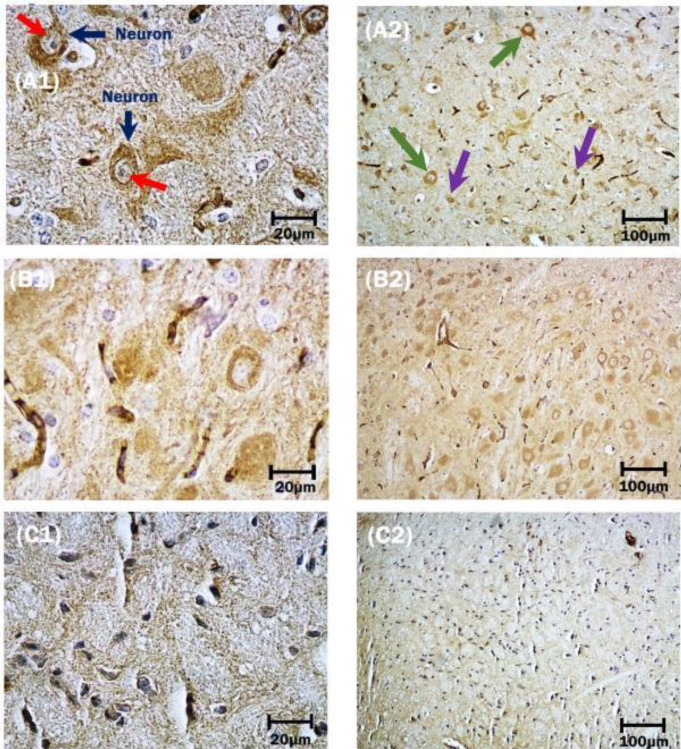
(A1 and A2): mixtures of small (violet arrows) and large-sized (green arrows) neurons are presented in surviving mice following intramuscular inoculation of street rabies virus. Nissl bodies (red arrows) in large neurons (dark blue arrows). These granules are the site of protein synthesis. (B1 and B2): IM-control group. (C1 and C2): show neurons in surviving IC-mice. Immunohistochemistry; 10X and 40X

As shown in Figure 6, large neurons in surviving mice injected by the IM route, are characterized by relatively large cell bodies (reticular formation), single nuclei with obvious nucleoli, and Nissl bodies (A1), which represent the rough endoplasmic reticulum. Immunoreactivity of neuronal cells with anti-MAP2 antibody was qualitatively more prominent in the brain slices prepared from surviving mice inoculated by IM than IC route of infection (A2). Qualitative observations were confirmed by statistically significant differences in the immunostaining results obtained by Image J and Prism software packages (*P*<0.0007) (Figure 4). Features of classic neuronal degeneration, including cell shrinkage, loss of Nissl substance, and small dark pyknotic cells were found in the brains of surviving mice inoculated by the IC route (Figure 6: C1 and C2). Among dead mice, although the number of neuronal cells demonstrated a significant decrease in both IC and IM inoculated mice, the number of this cell in IC-mice was less than in the IM group in surviving mice(*P*<0.0001) (Figure 4).

**Table 1:** Characteristics of CVS-11 and Street rabies virus (SRV) used for IM and IC inoculations

CVS-11 Dilutions injected intracerebrally (0.03 ml) LD50/dose:6.518							CVS-11 Dilutions injected intramuscularly (0.1 ml) LD50:1.714									
	$10^{-5}$	$10^{-6}$	$10^{-7}$	$10^{-8}$	$10^{-9}$	IC* Control	$10^{-1}$	$10^{-2}$	$10^{-3}$		$10^{-4}$		$10^{-5}$	$10^{-6}$	IM** Control	
	N=10	N=10	N=10	N=10	N=10	N=5	N=10	N=10	N=10		N=10		N=10	N=10	N=5	
**Mortality rate (%)**	100	87.5	14.3	0.0	0.0	----	100	35.0	0.0		0.0		0.0	0.0	----	
	Total number:55						Total number:65									
SRV Dilutions injected intracerebrally (0.03 ml) LD50/dose: 5.274							**SRV Dilutions injected intramuscularly (0.1 ml)** **LD50/dose: 2.400**									
	$10^{-3}$	$10^{-4}$	$10^{-5}$				$10^{-1}$	$10^{-2}$	$10^{-3}$	$10^{-4}$						
	N=10	N=10	N=10				N=10	N=10	N=10	N=10						
**Mortality rate (%)**	100	100	62.5				100	40.0	0.0	0.0						
	Total number:30						Total number:40									

LD_50_/dose: 50% effective lethal dose

IC*: Intracerebral, IM**: Intramuscular

**Table 2:** Incubation period of SRV compared with CVS-11 by intramuscular (IM) and intracerebral (IC) route of inoculations indicates a longer incubation period for street rabies virus by the two routes of infection

**Route of inoculation**	**SRV**	**CVS-11**
IM	11.7 days (CI: 10.48-12.94)	6.2 days (CI: 5.82-6.49)
IC	8.6 days (CI: 7.97-9.14)	5.0 days (CI: 5.0-5.0)

## Discussion

In this experiment, the prominent impact of strain type (street or laboratory-fixed) on mortality rate via IM inoculation suggests lower affinity of CVS-11 to its natural receptors at the nerve endings following IM inoculation which might result in poor infectivity of the virus (5); however, it is not still clear whether infection of muscle fibers is a basic step before accessing into the peripheral nervous system. Also, the lesser tendency of street rabies strains to induce pathogenesis than that fixed type is attributed to the neuroadaptation of fixed strains following serial passages in animal brains (29). Another possibility explaining resistance to SRV infection is caused by inefficient processing of viral proteins through host immune mechanisms or response to internal or non-structural viral proteins by the neutralizing antibody (30, 31).

In humans infected with SRV strains, inflammation of the CNS rarely occurs. However, some inflammatory changes, including increased microglial activation and astrocyte protein expression (GFAP) may be observed (26). Microglial cells are intracerebral resident macrophages, which represent 5–15% of brain cells, with dual roles of neuroprotection and detrimental scavenging function (32-34). Following injury, their morphology changes to the amoeboid type (34, 35) and produce different proinflammatory cytokines and chemokines, which cause nerve cell death and cytotoxicity via oxidative damage, leading to immunosuppression and pathogenesis in the brain(36). Microglial cells also induce neuroprotection by releasing neuroprotective substances (e.g., plasminogen) and IL-6 (37).

In the present study, the unexpected results with unchanged morphology of microglial cells are inconsistent with the results reported by Kimitsuki *et al.*(38) and represent the assumption that microglial cells and their inflammatory cytokines may not contribute to neuroprotection in the brain following intracerebral inoculation of SRV and survival of the mice. Moreover, in dead mice, both IC and IM inoculations of SRV showed an increased number of microglia which could be explained by that the infection of microglial and astrocyte with the rabies virus probably favors dysfunction or even death of these cells in dead mice (39).

Kesdangsakonwut *et al*. indicated that intrathecal immunization of mice infected with SRV may yield more robust protection and recovery than infections caused by the fixed strain (CVS)(40).

Astrocytes contribute to a variety of essential functions, especially pathological entities. Reactive astrogliosis is known as a sensitive marker of tissue damage and a gradual phenomenon, ranging from reversible changes in gene expression and cell hypertrophy to rearrangement of tissue structure and scar formation. It is also considered as one of the consequences of brain/spinal cord infection, injury to the CNS and retina, brain tumors, stroke, and neurodegenerative diseases (41-44). The prominent features of this activation include up-regulation of GFAP expression and hypertrophy of astrocyte processes (45). It has been suggested that microglial activation precedes astrocytic hypertrophy, leading to reactive astrogliosis (46, 47). In this study, the number of astrocytes increased following intramuscular inoculation of SRV in the dead group but did not change in surviving mice. Since astrocytes are known to be more resistant to the disturbed milieu of the brain, they may maintain the neuronal integrity in the surviving mice by regulation of pH and ions, removal of excitotoxins, scavenging of free radicals, and release of neurotrophins (48), while in dead mice, a greater degree of derangement(probably caused by heavier viral loads) results in astrocyte dysfunction, which aggravates the situation and promotes the spread of the virus throughout the brain. SRVs can also produce a persistent infection in astrocytes by evading the pathogen recognition receptors and cause failure in mounting a type I IFN response in astrocytes. Therefore, astrocytes cannot restrict the virus following neuronal transport to the CNS (20). In this regard, a study revealed differences in the up-regulation of an identified series of genes, including neuroligins and growth factors in astrocytes and neurons, which are associated with neuronal dysfunction and spread of infections caused by street and fixed rabies viruses in the mouse brain (39). Kimitsuki *et al*. reported more severe pathological changes in the dorsal root ganglion, compared with the nerve cells in the brain and spinal cord following IM inoculation of SRV, while here, different routes of SRV inoculation had no effects on the GFAP expression of astrocytes in the dead mice (38).

MAP-2 is an essential protein in the dendritic cytoskeleton, which plays a basic role in neuronal morphogenesis and stability maintenance of the dendritic tree, especially in the adult brain. Alterations in MAP-2 expression have been documented in rabies and many neurological diseases (49-52), although there is still little known about changes in this protein due to rabies. Researchers who used immunofluorescence with MAP-2 found no changes in neurons following rabies (53), but another study reported increased MAP-2 immunoreactivity in the cerebral cortex of mice inoculated with both fixed and SRV strains via two routes of inoculation (IM and IC) (21). In contrast, the reduced number of MAP2-positive cells in the dead mice, inoculated by both routes of injection, was shown in the present study, which could be due to the viral induction of neuronal death. Generally, dendritic pathology is correlated with the depletion of MAP-2 expression (52), although both decrease and increase of MAP-2 expression are correlated with neuronal pathology (50). During rabies infection, loss of the key function of astroglial cells results in neuronal dysfunction and death (48).

## Conclusion

Taken together, we concluded that SRV infection of mice induced innate cellular responses in the brain by which the number of functional astrocytes and microglial cells was not enough to affect disease development, neuroprotection, and recovery, following IC and IM inoculations in the dead group of mice. In addition, major neuronal loss by decreased MAP-2 expression in both surviving and dead mice might indicate the disintegration of neuronal cytoskeleton and imbalance in CNS milieu which aggravates the infection following SRV infection. Although we believe in the need for further investigations to elucidate the pathological mechanisms of street rabies strains and confirm the present findings.
